# Effectiveness of Tocotrienol-Rich Fraction in Older Adults: Protocol for a Randomized, Double-Blind, Placebo-Controlled Trial

**DOI:** 10.2196/73039

**Published:** 2025-09-23

**Authors:** Nor Amira Nabila Amir Razak, Jo Aan Goon, Wan Zurinah Wan Ngah, Suzana Makpol, Mohd Hanafi Ahmad Damanhuri, Nor Faeizah Ibrahim, Nur Fathiah Abdul Sani, Nuraqila Mohd Murshid, Anis Faqihah Mohd Azizan, Kok Yong Chin, Amilia Aminuddin, Mohd Heikal Mohd Yunus, Munirah Md Mansor, Juvenia Rui En Neo, Wei Ney Yap, Hsieu Yen Loong, Yee Wei Ung

**Affiliations:** 1 Department of Biochemistry Faculty of Medicine Universiti Kebangsaan Malaysia Kuala Lumpur Malaysia; 2 Department of Pharmacology Faculty of Medicine Universiti Kebangsaan Malaysia Kuala Lumpur Malaysia; 3 Department of Physiology Faculty of Medicine Universiti Kebangsaan Malaysia Kuala Lumpur Malaysia; 4 Department of Pathology Faculty of Medicine Universiti Kebangsaan Malaysia Kuala Lumpur Malaysia; 5 Research and Development Department Davos Life Science Singapore Singapore; 6 Research and Development Department KL-Kepong Oleomas Selangor Malaysia

**Keywords:** tocotrienol, skin, cognition, brain, weight, satiety, aging, oxidative stress, immune, inflammation

## Abstract

**Background:**

Tocotrienol, a naturally occurring form of vitamin E, has been extensively studied for its potent antioxidant, anti-inflammatory, and immune-stimulating properties. However, the clinical impact of tocotrienol supplementation on older adults’ overall health and well-being remains relatively unexplored. This research aims to investigate the efficacy of tocotrienol-rich fraction (TRF) on various health parameters associated with general well-being in individuals aged between 50 years and 75 years.

**Objective:**

It is hypothesized that TRF supplementation may exhibit positive outcomes on blood biochemistry and several physiological aspects, including lowered levels of oxidative stress and inflammation biomarkers; improvement in vascular age; and enhancement of skin condition, bone mineral density, and cognitive function.

**Methods:**

This randomized, double-blind, placebo-controlled trial was designed to investigate the effectiveness of TRF supplementation on overall health in healthy older adults. The study aims to assess the impact of a daily dosage of 200 mg of TRF over a period of 6 months. A total of 220 participants is enrolled in the study, with one-half receiving the placebo and the other one-half receiving TRF supplementation. The study comprises 3 time points: baseline, 3 months, and 6 months. At each time point, various measurements are taken to evaluate different aspects of health. The primary outcome measurements include blood biochemistry assessments, such as liver function tests, renal profile, lipid profile, and full blood count. Oxidative stress markers, including malondialdehyde, advanced glycation end products, protein carbonyl, and isoprostane, are also evaluated. Immune response markers such as interleukin-6 and tumor necrosis factor-α are assessed. Satiety regulation is examined through measurements of leptin and ghrelin. Body composition and skin health parameters, including wrinkling, pigmentation, elasticity, hydration, and sebum secretion, are evaluated. Additionally, arterial stiffness is assessed using arteriography at baseline and 6 months. For secondary outcome measures, bone mineral density is measured using dual x-ray absorptiometry, and cognitive function is assessed using the Montreal Cognitive Assessment, Rey Auditory Verbal Learning Test, and digital span test. Both bone mineral density and cognitive function are also measured at baseline and 6 months.

**Results:**

The study is progressing as planned, with 209 participants recruited as of April 2025. The research was funded in 2019, and data collection started in December 2020. Preliminary data analysis has been completed for the first 120 participants, and final results are expected upon completion of data collection and unblinding in 2026.

**Conclusions:**

By comprehensively evaluating these health aspects, this study seeks to provide valuable insights into the potential benefits of tocotrienol supplementation for promoting the overall health and well-being of the aging population.

**Trial Registration:**

National Medical Research Register (NMRR) NMRR-19-2972-51179; https://tinyurl.com/yy9yueer

**International Registered Report Identifier (IRRID):**

DERR1-10.2196/73039

## Introduction

### Background

Aging is a progressive process characterized by the gradual loss of tissue and organ function over time [1]. Two theories of aging include the free radical theory of aging, which later evolved into the oxidative stress theory of aging, and a theory based on low-grade inflammation, or inflammaging, in older adults. The oxidative stress theory of aging is based on the hypothesis that age-related functional declines occur as a result of the accumulation of oxidative damage to macromolecules such as lipids, DNA, and proteins caused by reactive oxygen and nitrogen species (RONS) buildup [2]. With advancing age, the accumulation of RONS leads to post-transcriptional modifications and oxidative damage, often contributed to by elevated levels of oxidative stress biomarkers including advanced glycation end products (AGEs), malondialdehyde (MDA), protein carbonyl (PC), and F2-isoprostanes (F2-IsoPs) [3]. In parallel, chronic low-grade inflammation has been linked to the onset of age-related chronic diseases such as cardiovascular disease, diabetes, and cancer [4]. In older adults, there is often an imbalance between excessive free radicals and low antioxidant levels, along with increased risk of chronic inflammation due to elevated inflammatory cytokine production, which may lead to disruption in normal cellular physiology [5,6]. High levels of oxidative stress biomarkers and inflammatory cytokines are associated with low cognitive performance, osteoporosis, atherosclerotic characteristics, skin conditions, and renal problems in older adults [7-9].

According to the Department of Statistics, Malaysia is officially regarded as an aging nation, as individuals aged 65 years and older were predicted to make up 7.7% of the population as of 2024 [10]. This indicates an urgent need to identify preventive, nonpharmacological strategies for maintaining the health and well-being of the older adult population. Introducing natural adjuncts like tocotrienol-rich fraction (TRF) supplementation may serve as a cost-effective and physiologically beneficial approach to reducing the health care burden associated with aging while potentially supporting healthy aging and improving overall quality of life for older adults.

Tocotrienol is a naturally occurring vitamin E that is predominantly found in palm oil [11]. It can be further distinguished into 4 isomeric forms (α, β, γ, and δ), depending on the location and number of methyl groups on the chromanol ring. Tocotrienols, as opposed to tocopherols, have unsaturated isoprenoid side chains with 3 double bonds, allowing for greater cellular penetration and dispersion inside lipid membranes [6,12]. This structural difference improves their antioxidant and anti-inflammatory properties. Cumulative scientific evidence has convincingly shown that tocotrienol possesses pronounced antioxidative function by effectively neutralizing free radicals, including RONS, which ultimately reduces oxidative stress [11,13-22]. Tocotrienol also possesses anti-inflammatory properties by ameliorating inflammatory markers that are consistently associated with age-related chronic diseases and disability [4], including interleukin-6 (IL-6), tumor necrosis factor-alpha (TNF-α), and C-reactive protein (CRP) [23-25]. IL-6 is a pleiotropic cytokine produced by the immune cells, vascular endothelial cells, adipocytes, and skeletal muscle and possesses both anti-inflammatory and pro-inflammatory properties [26]. TNF-α, another cytokine, is mainly produced by macrophages and other cell types in response to cell damage [27], while CRP is an acute-phase protein produced by the liver in response to elevated IL-6 and TNF-α levels [28-30]. These properties render tocotrienol an attractive therapeutic candidate for slowing down the aging process and thus potentially extending the lifespan of older adults [11,13].

Moreover, tocotrienol also modulates inflammation by targeting crucial transcription factors such as nuclear factor kappa B (NF-κB), the signal transducer and activator of the transcription-3 (STAT-3) pathway, and other cytokine regulators integral to the inflammatory signaling cascade [12,13,22,31-33]. Tocotrienols have been shown to exhibit isoform-specific effects on anti-inflammatory potency. For instance, several studies have demonstrated that δ-tocotrienol exhibited the most potent inhibitory effect on IL-6 and TNF-α production as compared with other isoforms [24,34]. Another study has shown that δ-tocotrienol inhibits TNF-α–induced NF-κB activation caused by upregulation of the anti-inflammatory protein A20 [35]. In addition, Muid et al [32] demonstrated that both δ- and γ-tocotrienols have higher potency in inhibiting IL-6 production and NF-κB activation than α- and β-tocotrienols in lipopolysaccharide-stimulated human umbilical vein endothelial cells. The NF-κB pathway inhibition induced by γ-tocotrienol also contributes to downregulation of oncogenic gene expression, thus promoting tumor cell apoptosis, which is crucial for suppressing tumor metastasis [36]. Moreover, combined treatment with γ-tocotrienol and an epidermal growth factor receptor inhibitor showed suppression in epidermal growth factor–dependent STAT-3 signaling in mammary tumor cells [37]. Collectively, these findings underscore the pivotal role of tocotrienols in modulating inflammation through multifaceted inhibition of key transcription factors, suppression of pro-inflammatory cytokines, and regulation of signaling pathways central to the inflammatory cascade. This positions tocotrienols as promising agents for the prevention and management of inflammation-driven diseases, including cancer and chronic inflammatory conditions.

Furthermore, these scientific investigations have illuminated tocotrienols’ ability to enhance skin health through UV protection and skin-lightening effects, both in preclinical and clinical models [38-42]. Tocotrienols have also demonstrated their potential for improving lipid profiles among individuals with dyslipidemia and hypertension [12,43-48] and have been shown to regulate leptin expression, thereby influencing appetite control and energy homeostasis [49-54]. To date, no study has specifically investigated the effect of tocotrienols on ghrelin expression. Therefore, our study aims to fill this knowledge gap and provide novel insights into this unexplored area.

In bone health, tocotrienols promote osteoblast survival and proliferation through free radical protection and inhibit osteoclasts by downregulating the mevalonate pathway. They also regulate gene expression to promote bone formation [55-66]. Similarly, tocotrienols positively influence neurological health by modulating gene expression in the brain, potentially enhancing memory and motor function while delaying Alzheimer disease progression. Notably, α-tocotrienol at nanomolar concentrations has been shown to attenuate enzymatic and nonenzymatic mediators of arachidonic acid metabolism and neurodegeneration [11,67-71].

TRF is a commercial mixture of vitamin E isomers, containing approximately equal proportions of α-tocopherol (αTP) and α-, β-, γ-, and δ-tocotrienol isomers. TRF supplement with a complete spectrum of tocotrienol isomers has been found to alleviate inflammatory symptoms of allergic rhinitis [72], enhance mental health and cognition [73], reduce inflammation and oxidative stress in ulcerative colitis [31], and mitigate UV-induced skin inflammation [38]. A previous study reported that TRF can improve antioxidant enzyme activities and glutathione levels in women aged between 50 years and 55 years [74]. In the same study, TRF supplementation was found to reduce MDA levels as early as 3 months, while oxidative damage toward DNA was reduced in women after 6 months of supplementation [75]. Furthermore, related isomers derived from the same source have demonstrated benefits in cardiovascular, liver, and metabolic functions [12,76] while exhibiting inhibitory effects on the growth of gastric cancer [77], prostate cancer [78], and breast cancer [79]. The findings from these studies provide a compelling basis to initiate a clinical trial aimed at evaluating the impact of TRF on the well-being of healthy individuals.

### Prior Work

To establish an appropriate dosage and duration for the clinical trial, systematic research of human studies involving vitamin E supplementation in healthy participants spanning the years 1997 to 2019 was conducted. A comprehensive search using keywords such as “human,” “vitamin E,” “tocopherol,” and “tocotrienol” in the PubMed database yielded 9 published papers. These studies shed light on the dosages and durations of tocotrienols that are suitable for this clinical trial. In a comparative study investigating the impact of supplementation with TRF and αTP on gene expression in healthy older adults, the administration of αTP (400 IU/day) and TRF (150 mg/day) over a period of 6 months was found to influence pathways related to immune response, drug response, cell adhesion, and signal transduction pathways. Notably, TRF supplementation exhibited a more pronounced effect than αTP in modulating gene expression involved in signaling pathways [80]. The same study also revealed that TRF and αTP supplementation demonstrated similar antioxidative and anti-inflammatory effects in older adults, with TRF showing more significant impacts in female participants [74]. This occurrence may be associated with the downregulation of the apoptotic pathway such as ERK1/2 cascades and NF-κB pathway after 6 months of TRF supplementation [80]. Moreover, another study revealed that TRF supplementation (150 mg/day) for 6 months led to alterations in plasma protein levels including upregulation of apolipoprotein A-I and E precursors and downregulation of CRP precursors in both young and older individuals, actions that are associated with a therapeutic effect against atherosclerosis [81]. These findings provide a perspective that TRF also alters protein expression as shown by elevated plasma protein levels following 6 months of TRF supplementation.

Another investigation focused on the effects of TRF supplementation at a dosage of 400 mg/day for 2 months on the immune response to tetanus toxoid immunization in healthy volunteers. The findings indicated that TRF exhibited immunostimulatory effects [82], thus reinforcing the notion that TRF is associated with handling cellular inflammation. In a randomized controlled trial spanning 6 months, daily supplementation of 150 mg/day TRF was associated with improvements in lipid profile and oxidative status in healthy older adults [83]. Interestingly, a separate trial found that daily supplementation with TRF (150 mg/day) for 6 months did not induce immunomodulatory changes in healthy human volunteers [84]. A much earlier clinical trial, comparing 50-mg with 100-mg vitamin E supplementation over 6 months, suggested that the higher dose (100 mg) had a more pronounced effect on cellular immune function in noninstitutionalized older adults aged 65 years to 80 years [85]. Moreover, a 4-month supplementation regimen of 60 IU to 800 IU vitamin E daily had no adverse effects on various health parameters, including general health, nutrient status, liver enzyme function, thyroid hormone concentrations, creatinine concentrations, serum autoantibodies, killing of *Candida albicans* by neutrophils, and bleeding time in healthy participants older than 65 years [86]. In another randomized controlled trial, older adults consuming 200 mg/d of vitamin E for 4 months exhibited an increase in delayed-type hypersensitivity skin response and an elevation in antibody titer to hepatitis B [87].

Following the review of the literature, a supplementation regimen of 200 mg per day for 6 months was selected. Although no published human studies have reported any serious adverse effects attributed to tocotrienols, it is worth noting that, in animal studies, tocotrienol doses of 500 mg/kg body weight and 1000 mg/kg body weight (administered orally) were found to increase bleeding and clotting times in mice during subacute (14 days) and subchronic (42 days) investigations [88]. When these doses are converted to a human equivalent dose, they equate to 2400 mg and 4800 mg, respectively in humans. This discovery implies that high doses of tocotrienols (>2400 mg) should be used cautiously, particularly by individuals with a tendency to bleed or those taking anticoagulants. Consequently, individuals who are currently on anticoagulant or antithrombotic medication are ineligible for participation in this research.

Although several human studies have explored the potential benefits of tocotrienols, there is a noticeable lack of published, comprehensive data that substantiate their advantages specifically in older adults. To address this knowledge gap, we designed a randomized, double-blind, placebo-controlled study, also widely recognized as the “gold standard” in intervention research [89]. This research design excels in establishing causal relationships, enabling us to demonstrate the efficacy of tocotrienols, compared with placebo, in older adults. The connection between oxidative stress, chronic low-grade inflammation, and the aging process, as well as age-related diseases, has been firmly established through extensive epidemiological studies involving older adults [90]. Consequently, several key investigative areas, including antioxidant and inflammation levels, arterial stiffness, appetite regulation, skin parameters, cognitive functions, and bone mineral density, were chosen as focal points for this research. Tocotrienol supplementation is predicted to have a positive impact on all of these parameters, adding to the increasing amount of data that demonstrates its ability to improve general quality of life of older adults. In particular, tocotrienols have been demonstrated to attenuate arterial stiffness [91], modulate hormones related to appetite [92], improve skin health through improvements in hydration and elasticity [93], promote cognitive performance through neuroprotective mechanisms [11,94], lower oxidative stress and pro-inflammatory markers [95,96], and positively influence bone metabolism [65].

The objective of this randomized, double-blinded, placebo-controlled study is to evaluate the efficacy of TRF supplement on health indices for older adults who have been reported to be at risk of reduced antioxidant levels and low-grade chronic inflammation, factors that contribute to an increased susceptibility to chronic diseases [97]. This study design offers the advantage of establishing causality by assessing the efficacy of TRF compared with placebo in older adults. The outcomes of this trial will provide crucial insights into the potential benefits of TRF supplements for enhancing the health and well-being of older adults.

### Aim of This Study

The comprehensive objectives of this research involve the evaluation of the effectiveness of TRF at enhancing antioxidant levels and reducing inflammation among individuals aged 50 years to 75 years. Furthermore, changes in appetite hormones, arterial stiffness, and skin parameters over 3-month and 6-month supplementation periods are monitored relative to baseline measurements. In addition, alterations in bone mineral density following 6 months of tocotrienol supplementation are being determined, and potential enhancements in cognitive function between baseline and the 6-month point are being investigated. Finally, the influence of supplementation on vitamin E levels within the body is being assessed after 3 months and 6 months of TRF supplementation.

## Methods

### Study Setting

Healthy men and women aged between 50 years and 75 years are recruited from the Klang Valley, which is centered in the federal territories of Kuala Lumpur and Putrajaya in Malaysia. Participants are divided into two cohorts, which are supplemented with either placebo or tocotrienols for 6 months.

### Ethical Considerations

This research upholds the principles of Good Clinical Practice and adheres to the pertinent regulatory guidelines established by the Malaysia Ministry of Health. The research was approved by the Research Ethics Committee of Universiti Kebangsaan Malaysia (ethics approval number: UKM PPI/111/8/JEP-2019-798) and was registered with the Malaysia National Medical Research Register and National Pharmaceutical Regulatory Agency. Funding for the study was provided by KL-Kepong Oleomas Sdn Bhd, with the research itself being independently conducted by scholars and experts from a higher learning institution. Before the clinical trial commenced, the two parties signed a distinct legal agreement designed to safeguard the rights of the sponsor and researchers involved.

The investigators have no financial nor other competing interests in relation to the overall trial, ensuring impartiality and scientific integrity. Any alterations to the protocol are communicated by the investigators to both the Research Ethics Committee and the sponsor prior to implementation.

Upon completion of the trial, the principal investigator (PI) and sponsor will collaborate in publishing the results and deriving pertinent conclusions while ensuring strict confidentiality of participants’ personal information.

The PI is responsible for retaining the data for a minimum duration of 7 years from the date of closure of the database. The sponsor possesses exclusive rights to use the data for commercial purposes, except for any personal information related to the participants. Concurrently, researchers are granted the privilege to use the data for noncommercial purposes, encompassing but not limited to patient care and treatment, academic pursuits, and publication.

Special ethical considerations have been addressed in accordance to the specific needs of the older adult participants in this study. Given the possibility of age-related cognitive decline, the informed consent process incorporates simplified language and comprehension testing to assure accurate understanding. Extra care is taken to reduce any psychological pain or stigmatization, and cognitive examinations are carried out using validated instruments suitable for older adults. All study procedures are intended to accommodate physical or emotional weaknesses, including flexible scheduling and participant comfort during data collection. Throughout the research process, these safeguards maintain each participant's autonomy, dignity, and safety. The eligible participants were compensated RM 40 (US $9.48) for each visit.

### Eligibility Criteria

Participants in this research must meet specific health criteria, including being generally healthy as determined using physical examination and lab-based blood tests, with satisfactory liver and renal function tests. Eligible participants can be of either gender and aged between 50 years and 75 years. They should not be allergic to palm oil and vitamin E and should not have consumed vitamin E or any other antioxidant supplements in the past 3 months. Furthermore, they must be willing and able to adhere to the study’s visit schedule and procedures, reside within a geographically feasible proximity for adequate follow-up (as determined by the investigator), and have a clear understanding of the study protocol, as indicated by their signed informed consent forms.

Participants in this study are expected to reflect greater distribution among the Malay and Chinese population, as these two races are the majority in Malaysia. Meanwhile, participation from the Indian community is less expected as they avoid bovine-related products in their dietary consumption due to religious reasons, aside from being the minority in the country. Moreover, the gender distribution is expected to be balanced between both men and women as there were 2 million individuals of each gender in people aged 60 years and older as of the 4th quarter of 2024 [10]. The socioeconomic background of the participants (ie, education background, occupation, and social status) is not entirely restricted as we intend to be inclusive of all population groups, aside from factoring in the limited availability of participants. However, we note that the participants’ education background may influence cognitive performance before and after supplementation. Thus, we will record their academic background when performing the assessments for cognitive function.

Participation in this research is restricted based on several exclusion criteria, such as individuals with fat malabsorption. This is because tocotrienols, as fat-soluble compounds, require adequate lipid absorption to achieve maximum bioavailability. In addition, those with chronic conditions such as cardiac diseases, neurological diseases, diabetes, HIV infection, or psychiatric illness or social situations are not eligible because these conditions and their associated treatments may have an impact on the study end points by obscuring the specific effects of tocotrienol supplementation.

Individuals following a vegan diet are also excluded due to potential nutritional differences, particularly in fat intake and fat-soluble vitamin levels, which may alter tocotrienol absorption and metabolism. Moreover, those who have smoked within the past 3 months are also excluded since smoking can cause oxidative and systemic stress, which can alter overall inflammation, vascular function, and antioxidant status. Excluding people who have recently undergone or are planning for surgery may be helpful to eliminate confounding side effects from surgical stress and postoperative drugs. Furthermore, pregnant and lactating women are excluded for ethical and safety reasons, given the lack of data on tocotrienol use in these populations. Individuals with a history of bleeding disorders, liver dysfunction, or gastrointestinal ulcers (eg, thrombocytopenia, abnormal liver function, liver diseases such as chronic hepatitis, gastrointestinal ulcers) are also excluded due to the potential risk of bleeding complications as well as reduced tocotrienol metabolism and clearance. Participants who are taking antibiotics, anticoagulants, antithrombotics, or other supplements that may interact with tocotrienol activity are excluded to avoid pharmacological interactions that could compromise research safety or supplementation efficacy.

### Interventions

This research encompasses the evaluation of two investigation products that are supplied by KL-Kepong Oleomas Sdn Bhd. The first product, known as DavosLifeE3 Complete (50 mg), contains 50 mg of tocotrienols that are sourced from palm oil and meets the specifications outlined in the US Food and Drug Administration “generally recognized as safe” exemption claim 21 CFR 170.36 (c) [98]. The second product, Placebo Softgel, is a palm oil supplement that has the vitamin E compounds stripped off, serving as a vital control within the study. The blinding of the supplement is performed such that each group is labelled with their respective intervention product (IP) number without disclosing the content in the softgel. The softgels for both groups are odorless, of similar size and shape, and packaged in a fully opaque bottle. Contract research assistants generated the random allocation sequence, enroll participants, and assign participants to the interventions. Study participants, researchers, and data analysts will remain blinded throughout the clinical study to maintain blinding integrity throughout the 6-month intervention period.

### Outcomes

The study outcomes are divided into primary and secondary end points. The primary end points include vascular health (arterial stiffness), blood biochemistry (liver function, lipid profile, renal function, full blood count, and fasting glucose), oxidative stress (F2-IsoPs, MDA, AGEs, and PC), inflammation markers (TNF-α and IL-6), appetite regulation (ghrelin and leptin), and skin assessment (elasticity, hydration, transepidermal water loss, pigmentation, sebum secretion, wrinkles, and roughness). Meanwhile, cognitive function (Montreal Cognitive Assessment [MoCA], Rey Auditory Verbal Learning Test [RAVLT], and digit span test) and bone density fall under the secondary outcome measures.

**Figure 1:**
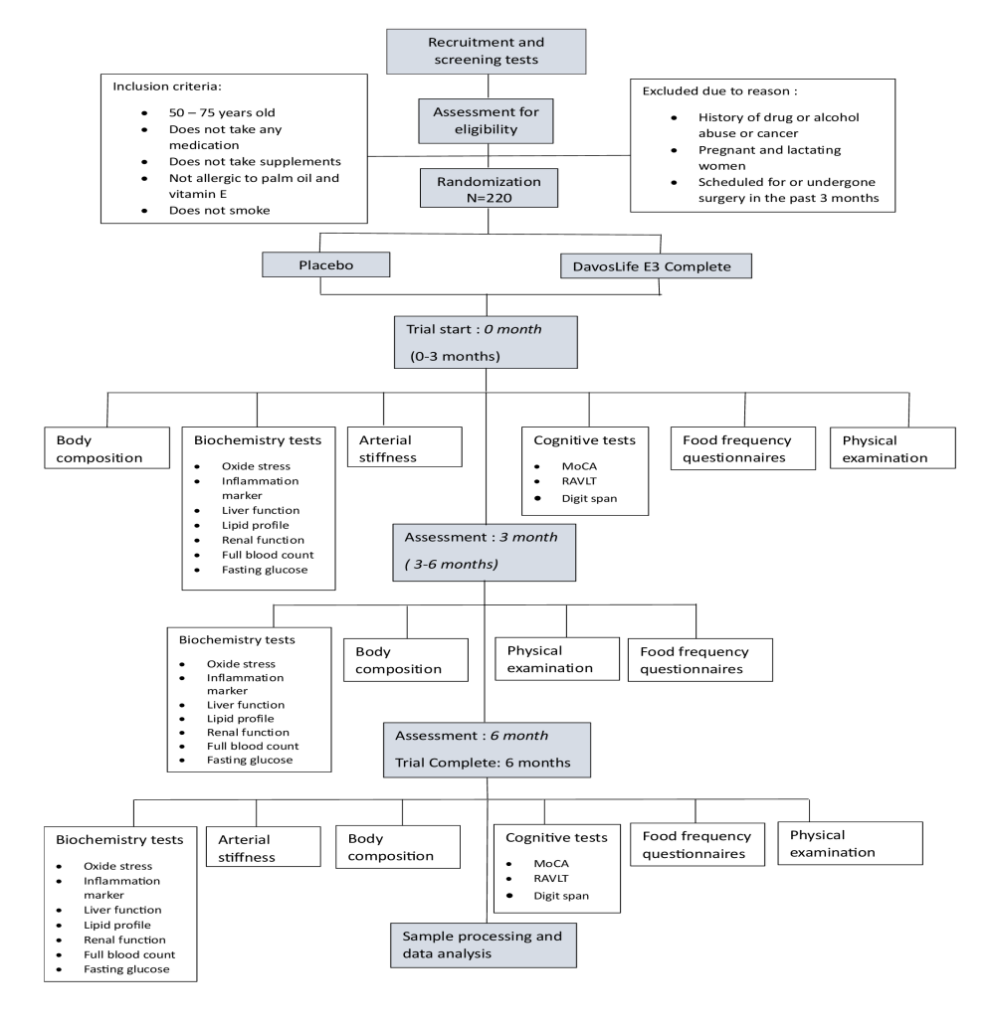
Overview of assessments and procedures for the 6-month clinical trial. DEXA: dual-energy X-ray absorptiometry; MoCA: Montreal Cognitive Assessment; RAVLT: Rey Auditory Verbal Learning Test.

The study encompasses a multifaceted array of assessments and procedures (Figure 1). For primary outcome measures, blood samples (15 mL) are collected as baseline samples for blood biochemistry analyses. Venous blood samples are drawn from the antecubital vein between 8 AM and 10 AM, following an 8- to 10-hour overnight fast. The standardized timing and fasting procedure are followed throughout all study visits (baseline, 3 months, and 6 months) to reduce biological variability caused by circadian rhythms and postprandial effects. After at least 5 minutes of rest to stabilize hemodynamic parameters before sampling, participants are seated, and blood is collected using sterile procedures.

Tubes containing samples are labeled with the participant’s code, trial ID, and date for accurate identification. From the collected blood samples, 4 mL is routed to the chemical pathology laboratory for blood biochemistry analysis. The remaining samples are stored appropriately on ice or in a 4 °C refrigerator until they undergo centrifugation, facilitating the isolation of plasma samples. These collected plasma samples are apportioned into 1-mL storage tubes and immediately stored in a –80 °C freezer. This storage method ensures that the samples are preserved for the analysis of oxidative stress markers (MDA, AGE, F2-IsoPs, and PC), inflammatory markers (TNF-α and IL-6), vitamin E, and appetite regulation markers (leptin and ghrelin), all to be conducted within 12 months of storage (Table 1).

**Table 1:** Summary of tests performed at 0, 3, and 6 months.

Number and time points, category, and parameters			Sample		Methodology	
**(1) Screening**						
	**Blood biochemistry tests**					
		Liver function (ALT^a^, ALP^b^, albumin, protein, bilirubin)	Blood		Clinical chemistry analyzer	
		Lipid profile (TG^c^, HDL^d^, LDL^e^, cholesterol)	Blood		Clinical chemistry analyzer	
		Renal function (Na, K, Cl, urea, creatinine)	Blood		Clinical chemistry analyzer	
		Full blood count (WBC^f^, RBC^g^, hematocrit, MCV^h^, platelets)	Blood		Clinical chemistry analyzer	
		Fasting glucose	Blood		Clinical chemistry analyzer	
		C-reactive protein	Blood		Clinical chemistry analyzer	
	**Physical examination**					
		Blood pressure	—^i^		Blood pressure monitor	
		Heart rate	—			
		BMI	—		Body weight scale and stadiometer	
	**Past medical history**					
		Medications	—		Screening form	
		Supplements	—		Screening form	
		History of illness	—		Screening form	
	**Social history**					
		Smoking	—		Screening form	
		Alcoholic consumption	—		Screening form	
		Exercise	—		Screening form	
		Dietary patterns	—		Screening form	
**(2) 0, 3, and 6 months**						
	**Physical examination**					
		Blood pressure	—		Blood pressure monitor	
		Heart rate	—			
		BMI	—		Body weight scale and stadiometer	
	**Body composition measurements**					
		BMI	Whole body		Body composition analyzer	
		Segmental and visceral fat	Whole body		Body composition analyzer	
		Basal metabolic rate	Whole body		Body composition analyzer	
	Blood biochemistry tests			Blood		Clinical chemistry analyzer
	**Oxidative stress markers**					
		AGEs^j^	Blood		ELISA^k^ kit	
		PC^l^	Blood		ELISA kit	
		F2-IsoPs^m^	Blood		ELISA kit	
		MDA^n^	Blood		HPLC^o^	
	**Inflammation markers**					
		TNFα^p^	Blood		ELISA kit	
		IL-6^q^	Blood		ELISA kit	
	**Weight management**					
		Ghrelin	Blood		ELISA kit	
		Leptin	Blood		ELISA kit	
	Vitamin E level			Blood		HPLC
	**Skin assessment**					
		Elasticity	Face and arm		Cutometer	
		Hydration	Face and arm		Corneometer	
		Transepidermal water loss	Face and arm		Tewameter	
		Pigmentation	Face and arm		Mexameter	
		Sebum secretion	Face and arm		Sebumeter	
		Wrinkles and roughness	Face and arm		Visioscan	
**(3) 0 and 6 months**						
	Arterial stiffness assessment			Whole body		Arteriograph device /photoplethysmography
	Bone density assessment			Whole Body		Dual-energy x-ray absorptiometry
	**Cognitive function assessment**					
		MoCA^r^	—		Questionnaires	
		RAVLT^s^	—		Questionnaires	
		Digit span test	—		Questionnaires	

^a^ALT: alanine transaminase.

^b^ALP: alkaline phosphatase.

^c^TG: triglycerides.

^d^HDL: high-density lipoprotein.

^e^LDL: low-density lipoprotein.

^f^WBC: white blood cells.

^g^RBC: red blood cells.

^h^MCV: mean corpuscular volume.

^i^Not applicable.

^j^AGEs: advanced glycation end products.

^k^ELISA: enzyme-linked immunosorbent assay.

^l^PC: protein carbonyl.

^m^F2-IsoPs: F2-isoprostanes.

^n^MDA: malondialdehyde.

^o^HPLC: high-performance liquid chromatography.

^p^TNFα: tumor necrosis factor-α.

^q^IL-6: interleukin-6.

^r^MoCA: Montreal Cognitive Assessment.

^s^RAVLT: Rey Auditory Verbal Learning Test.

Furthermore, the assessment of arterial stiffness is conducted by skilled laboratory assistants using arteriography during the participants’ visits at both the 0- and 6-month time points. This assessment uses inflatable cuffs and is noninvasive. The arteriograph primarily measures pulse wave velocity, augmentation index, and central blood pressure, all of which reflect vascular health. Total body fat and lean mass measurements are accomplished using an InBody device during the participant visits at 0, 3, and 6 months.

In addition, skin health is analyzed using a Cutometer (Courage+Khazaka electronic GmbH) and Visioscan (Courage+Khazaka electronic GmbH) during participant visits at 0, 3, and 6 months. At 2 anatomical locations (volar side of the forearm and lateral periorbital region of the face), skin characteristics such as elasticity, hydration, transepidermal water loss, pigmentation, sebum secretion, wrinkles, and roughness will be studied. At both locations, 4 to 5 repeated measurements are made for each parameter, and the results are averaged. To ensure site uniformity and accommodate anatomical changes, slight positional deviations of a few centimeters are permitted. By measuring at the forearm and face, it is possible to conduct a thorough analysis of skin alterations in regions with low and high levels of sun exposure, respectively. This increases sensitivity to both the local and systemic effects of the intervention.

Subsequently, for secondary outcome measures, participants undergo cognitive function assessments, which include the MoCA, RAVLT, and digit span test. These assessments are performed with the assistance of a trained research assistant during the participants’ visits at 0 and 6 months. Finally, the measurement of bone mineral density is conducted using a dual-energy X-ray absorptiometry device at 0 and 6 months. Full body, lumbar spine, and hip scans are conducted. The lumbar spine and hip are essential locations for measuring bone density in older adults, which is crucial for comprehending how tocotrienol supplementation affects bone health. These regions are significant to the study since they represent general bone health.

To monitor changes in dietary habits, participants complete a food frequency questionnaire during their visits at 0, 3, and 6 months. The food intake checklist was developed to monitor changes in dietary habits throughout the study. Nutritionist Pro software is used to analyze the diet history data for each participant to quantify their nutrient intake values. Statistical analysis is then conducted to determine whether the nutrient value is significantly different from the blood biochemistry composition. Figure 1 presents a flowchart illustrating the study period and the tests conducted at each time point.

### Participant Timeline

Over the study period, 3 assessments are carried out at 0, 3, and 6 months across various domains of inquiry, encompassing oxidative stress, inflammation, skin conditions, arterial stiffness, cognitive function, appetite regulation, bone mineral density, and vitamin E levels. To ensure comprehensive oversight of the assessments, delegation of an authority log was established and formally endorsed by the respective investigators, each of whom possesses expertise in their specific area of assessment.

### Sample Size

Sample size estimation was based on a comparison of mean low-density lipoprotein levels, referencing a previous case-control study on biomarkers for premature coronary artery disease by Shukor et al [99]. The sample size was calculated using a power and sample size program. The effect size was calculated based on a mean value of 3.11 (SD 0.81) for group one and a mean value of 2.66 (SD 1.25) for group two.

The total number of required participants calculated per group of the experimental design was 93 when dichotomous outcomes were presumed with a Type I error probability, α of .05, and power of 0.8. To maintain the power, a 20% dropout rate was factored in. Consequently, 110 participants are required to be enrolled in each group (placebo and supplementation). Therefore, a total of 220 participants are required to perform this clinical study.

### Recruitment

The selection of potential trial participants is conducted by the PI based on the inclusion and exclusion criteria. Participants who fail to meet the inclusion and exclusion criteria are defined as screening failures. The PI maintains a Screening Log, which includes screen failures to ensure a systematic selection of participants. Informed consent is obtained from each participant by the contract research assistants. During enrollment, general information (name, gender, age, address, and contact information) is obtained. To ensure security and confidentiality, each participant is assigned a participant-specific code. The code is used to label the participant’s laboratory results. Each participant is reimbursed for standard transportation fees.

Participants are free to withdraw from the study at any time for any reason. Participants may also be withdrawn at any time at the discretion of the PI. Possible reasons for withdrawal include abnormal laboratory values, protocol violation, the participant requiring the use of an unacceptable concomitant medication, participant not complying with protocol procedures, participant developing a condition during the study that violates the inclusion or exclusion criteria, loss to follow-up, death, or any other reason in the PI’s opinion that would impede participation in the study.

### Assignment of Interventions

Tocotrienols and placebo supplements are prepared and labelled with the IP number by the sponsor. Assignment of supplements to participants is by chance through the drawing of lots by contract research assistants. The treatment is double-blinded throughout the study period until all data have been collected and analyzed, after which the supplementation IP numbers are revealed to the PI by the sponsor. However, in case of any serious adverse event reported during the study period, the unmasking of the code is immediate.

A set of numbers, from 1 to 60, is randomized using the randomization application for every block of 60 participants. The participants are assigned a randomized number according to the numerical sequence 1 to 60 in the list of randomized numbers. All even numbers are treated with the same IP, while odd numbers are treated with another IP. Thus, every block randomization has an equal number of participants for each IP. The randomization will be repeated until 220 participants are recruited.

The unblinding in emergency situations is only permitted in case of a suspected, unexpected serious adverse reaction or other important adverse event, when knowledge of the supplemented product in question is required for therapeutic decisions in the medical management of the participant.

In the event of an emergency, an emergency decoding option is accessible to both the PI and designated personnel at the sponsor. The responsibility for breaking the blind for individual participants in emergency situations lies with the PI. If the need to break the blind arises, the PI will communicate this to the sponsor. Before the treatment code is broken, the PI responsible for unblinding must document the reason and date for unblinding in the case report form (CRF) to ensure proper documentation of the unblinding event, including a clear explanation of the reason for unblinding.

In the event that the sponsor requires unblinding of the treatment, the reason and date of unblinding are recorded in the CRF. In situations where it is necessary to unblind an individual participant’s treatment for the purpose of expedited reporting to the authorities, only designated individuals at the sponsor who are responsible for reporting this information will have access to the identity of the product. Every effort is made to ensure that all other members of the clinical trial remain blinded throughout the entire duration of the trial.

### Data Collection and Management

During the screening process, potential participants are assessed for eligibility to enter the study according to the inclusion and exclusion criteria. After an overnight fast, a 10-mL blood sample is taken from each volunteer. The screening tests consist of comprehensive evaluations, encompassing blood lipid profile, liver function tests, renal profile, full blood count, and fasting blood glucose measurements. Additionally, records of past supplementation and medical history are reviewed to obtain a holistic understanding of the individual’s health status. The results of blood tests are revealed to volunteers upon request. Volunteers who meet the inclusion criteria are selected for baseline sampling typically within a timeframe of 1 month to 2 months after the screening process.

Eligible participants are contacted to schedule appointments for data collection at baseline, 3 months, and 6 months. During each visit, a physician conducts a comprehensive medical evaluation, encompassing an assessment of medical history; documentation of any concurrent medications; measurements of participants’ weight, height, resting blood pressure, pulse rate, and temperature; and evaluation of vital signs. Subsequently, blood samples are drawn from each participant for immediate analysis of blood biochemistry, and additional samples are preserved for future analysis of oxidative stress markers, inflammatory markers, vitamin E, and appetite regulation marker levels. Furthermore, on the same day as the visit, assessments are performed for arterial stiffness, body fat composition, skin health, bone mineral density, and cognitive function.

Participant records and data for analyses are kept in separate folders according to the protocol and transferred into the CRF of each participant by the contract research assistants. The result for each assessment is monitored by specific investigators with expertise in the field, to facilitate comprehensive monitoring of the data.

A contractual disclosure agreement signed by the sponsors and investigators outlines the sponsor’s access rights to the trial data set and source data. Both the investigators and sponsor should collaborate and jointly author publications reporting the trial’s results to ensure an accurate representation of the results and drawing of appropriate conclusions, thereby preserving the integrity of the findings. All data are reported in a collective manner with no reference to any participants in the trial to ensure that the identities of participants remain confidential.

### Analyses

Statistical analysis is conducted using SPSS (IBM Corp). The Kolmogorov-Smirnov test will be performed to assess the normality of the data distribution. Type III sum of squares is set to evaluate the hypotheses.

A mixed-design ANOVA will be used to determine whether supplementation affects the blood biochemistry parameters (inflammation and oxidative stress) and physical health (body composition, skin, cognitive function, and vascular health) at baseline, 3 months, and 6 months among the groups. The Mauchly test of sphericity will be conducted to assess homogeneity of variance and compare the means of the supplemented group with those of the placebo group. To account for the risk of a Type I error as a result of many primary and secondary outcomes, multiplicity correction procedures will be used. In particular, the Benjamini-Hochberg approach for false discovery rate control will be used in each outcome domain (eg, oxidative stress markers, inflammatory markers, cognitive tests). Statistical power and Type I error control are balanced for exploratory research in this method with connected variables. The Tukey post hoc test will be used for comparisons between groups, while the Dunnett test will be used for comparisons within groups over time. In terms of missing data, linear mixed-effects models with the missing at random assumption will be used, which is acceptable given the repeated-measures design and inclusion of observed predictors. Nevertheless, dropout rates in older adult groups might differ from missing at random (for example, because of undetected health decline). To evaluate the possible impact of data missing not at random, sensitivity analyses will be conducted using pattern-mixture models and multiple imputation with delta-adjustment scenarios. These methods aid with determining the robustness of the findings under alternate missingness assumptions. All data will be presented as mean (SD), with statistical significance defined as *P*<.05.

This study requires a total of 220 volunteers as calculated using the power and sample size program. In the case where any participant withdraws or is eliminated during the course of the project, their ID number is not reused. Instead, new volunteers are recruited to maintain the total sample size at 220. The distribution of the IP numbers is rerandomized and adjusted as needed to ensure that both groups remain equally balanced, with 110 participants receiving each IP.

All randomized participants are included in the primary analysis using the intention-to-treat approach, which analyzes participants based on their initial group allocation regardless of whether they adhere to the intervention. Only participants who complete the study without significant protocol deviations are included in a secondary per-protocol analysis.

### Monitoring

Participants are instructed to maintain their regular lifestyle throughout the study duration. We recommend that they adhere to their usual routine regarding the number of meals and dietary plan. If there is a need to use any medication, they are required to inform the PI to ensure proper documentation in the CRF.

To ensure compliance, participants are requested to submit the provided supplement bottles during their visits at 3 and 6 months. The percentage of the leftover supplements in the returned bottles are used to track compliance. The purpose of these data is to evaluate intervention adherence. To account for adherence-related differences in the treatment effect, the compliance percentage is incorporated as a covariate in the statistical analyses. Additionally, subgroup analyses based on adherence levels (high, medium, and low compliance) will be carried out.

Additionally, plasma vitamin E levels are measured at 0, 3, and 6 months to assess the participants’ response to supplementation. In the event of suspected side effects or adverse reactions, participants should promptly report to the PI and seek immediate medical assistance. The PI must assess whether the observed effects are related to the trial and determine if the participant should be excluded from the study accordingly.

## Results

The study was approved by the Research Ethics Committee of Universiti Kebangsaan Malaysia for implementation from January 27, 2020, to July 26, 2026. The sponsor successfully disbursed funding in June 2020. Participant screening, recruitment, and data collection commenced in September 2020. As of April 2025, 339 participants were screened, with 211 recruited and 167 having completed the study. Data analysis was completed for the first 120 participants, evenly divided between the placebo and TRF supplementation groups. Final results are expected to be published in 2026, following the completion of data collection and unblinding of the IPs by the sponsor.

## Discussion

This study was conducted to evaluate the effectiveness of TRF (DavosLife E3 Complete) on blood biochemical composition (liver function, lipid profile, renal function, full blood count, and fasting glucose), oxidative status biomarkers (MDA, AGE, F2-IsoPs, and PC), inflammation biomarkers (TNF-α and IL6), appetite regulation biomarkers (leptin and ghrelin), arterial stiffness, skin health, bone density, and cognitive function in healthy older adults aged 50 years to 75 years over a 6-month intervention period. We hypothesized that the high antioxidant and anti-inflammatory properties of TRF would result in significant improvements across several different areas, particularly vascular function and cognitive performance. In order to provide more thorough knowledge on the role of TRF in promoting healthy aging, skin and bone parameters were also included to investigate the potential benefits of TRF on structural and aesthetic elements of aging. The goal of this study is to meet the increasing need for a safe, nonpharmacological intervention that can help the older population maintain their functional lifespan, quality of life, and physiological resilience.

The 5-year clinical trial was designed in accordance with the SPIRIT (Standard Protocol Items: Recommendations for Interventional Trials) guidelines and registered in the National Medical Research Register of Malaysia. Prior to the initiation of the trial, the investigators and sponsor signed a mutual confidentiality agreement. This agreement serves to safeguard against any unauthorized use, disclosure, publication, or dissemination of confidential information by both parties involved. Separately, a comprehensive research agreement was signed, addressing the roles of the investigators and sponsor, ownership of liabilities, indemnification and insurance, quality assurance, record-keeping, reporting, access, intellectual property, and publication and authorship.

The trial commenced in 2019 after ethical approval was obtained. Notably, although sponsored by a commercial company, the trial is conducted entirely by the institution independently of the sponsors. Participant recruitment, sampling, and analyses are performed by the investigators who are blinded to the participants’ identification to prevent bias. Volunteers undergo random screening and are recruited based on their fulfillment of the inclusion criteria. Similarly, the administration of investigational products is also randomized.

The trial protocol was collaboratively designed by investigators from diverse backgrounds, each bringing their expertise in the relevant measured parameters. Through careful consideration and discussion, the team determined the optimal parameters to be measured and the appropriate time points at which these measures were most likely to effectively detect changes. As a result, blood tests, skin assessment, and body composition were assessed at 3 time points, while parameters such as bone density, arterial stiffness, and cognitive function were measured exclusively at the baseline (0 months) and end point (6 months) evaluations.

The MoCA was used under the licensing agreement with the MoCA Clinic and Institute, ensuring that only trained research assistants administered the test to the participants. The RAVLT is a widely recognized measure that assesses an individual’s capacity to encode, integrate, retain, and retrieve verbal information across various stages of immediate memory [100]. As a result, this assessment tool is valuable for evaluating the impact of interference stimulus, delayed memory, and recognition. Additionally, the digit span test, which gauges the ability to maintain and manipulate a limited amount of information within a readily accessible state for short durations, typically less than 30 seconds, provides crucial insights when assessing working memory [101].

The dosage of TRF was determined based on previous reports that demonstrated its efficacy while not reporting any adverse events. Careful consideration was given to selecting an appropriate dose to ensure safety and prevent potential toxicity, as TRF is a fat-soluble compound and excessive dosage can lead to adverse effects. Research with rats has shown that oral administration of a vitamin E supplement was not safe for the liver and kidney [102]. Nevertheless, considering the ongoing changes in population lifestyles and dietary habits, it becomes imperative to conduct a comprehensive review of the TRF dosage for future clinical trials especially for therapeutic purposes.

On average, the screening process typically lasts around 2 hours. According to the latest data, approximately 60% of the volunteers successfully met the inclusion criteria and became eligible for recruitment into the study. The primary reasons for volunteer exclusions were elevated levels of total cholesterol, low-density lipoprotein cholesterol, fasting blood glucose, and blood pressure. On average, each participant requires approximately 3 hours to complete all assessments, excluding blood tests. To accommodate the fasting requirement for blood withdrawal and arterial stiffness assessment, these tests are conducted first. Subsequently, the remaining assessments are performed simultaneously at different stations, allowing participants to visit any available station. To minimize the waiting time, a maximum of 5 participants are scheduled per sampling day. This ensures that participants do not experience prolonged waiting periods.

Throughout the treatment period, the research assistants monitor the compliance of the participants through regular phone calls and short messages. However, the effectiveness of this monitoring method relies on the participants’ availability to respond to or read the messages. Noncompliant cases were primarily attributed to participants’ difficulty with remembering to take the supplement, which is evident when they return the bottles with a higher number of remaining softgels than expected during the 3- and 6-month visits. The data from noncompliant participants are still analyzed. However, it is specifically noted in the CRF so that any potential outliers in data analysis can be identified later.

The findings of this study will further extend our current knowledge in many ways. As the older adult population continues to increase globally [103], understanding the efficacy of TRF supplementation for promoting healthy aging becomes increasingly imperative. The use of a randomized, double-blind, placebo-controlled study protocol to assess the effectiveness of TRF in older adults offers numerous advantages that significantly enhance the credibility and reliability of the research findings. First, the randomization process ensures an unbiased allocation of participants into the treatment and control groups, minimizing the influence of confounding variables and increasing the validity of the study results. Second, the double-blinded nature of the trial eliminates both participant and researcher bias, as neither knows who is receiving the active intervention or the placebo, thereby preventing any preconceived notions from influencing outcomes. This rigorous blinding protocol ensures the integrity of the data collected and provides a more accurate assessment of the true effects of TRF supplementation. Finally, the inclusion of a placebo group enables researchers to distinguish between the specific effects of TRF and any placebo effects, ensuring that observed improvements in health outcomes can be confidently attributed to the intervention. Overall, by adhering to such a meticulous study protocol, researchers can generate robust evidence regarding the efficacy of TRF for promoting health and well-being among older adults, ultimately informing clinical practice and guiding future research endeavors.

There are some possible limitations of this study. One significant limitation is the potential for dropout or noncompliance among older participants, which can compromise the integrity of the study results and introduce bias. Older adults may face unique challenges such as cognitive impairment, mobility issues, or comorbidities that affect their ability to consistently adhere to the study protocol. Moreover, the relatively long duration often required for such trials increases the likelihood of attrition over time. Additionally, the strict inclusion and exclusion criteria necessary to ensure the internal validity of the study may limit the generalizability of the findings to broader older adult populations with diverse characteristics and health profiles [104]. Thus, researchers must remain cognizant of these limitations and use strategies to mitigate their impact on the study outcomes.

In conclusion, our study holds immense promise for advancing our understanding of the potential health benefits of TRF. While acknowledging the inherent challenges and limitations associated with conducting trials in older adult populations, the insights gleaned from this study have the potential to significantly impact clinical practice. Ultimately, by generating robust evidence regarding the efficacy of TRF supplementation for promoting healthy aging, this study protocol has the potential to contribute valuable insights to the field of geriatric medicine, paving the way for improved strategies to enhance the quality of life and well-being of older adults worldwide.

## Appendix

Multimedia Appendix 1 SPIRIT (Standard Protocol Items: Recommendations for Interventional Trials) checklist.

## Abbreviations

αTP: alpha tocopherol
AGE: advanced glycation end product
CRF: case report form
CRP: C-reactive protein
DEXA: dual-energy X-ray absorptiometry
F2-IsoPs: F2-isoprostanes
IL-6: interleukin-6
IP: intervention product
MDA: malondialdehyde
MoCA: Montreal Cognitive Assessment
NF-κB: nuclear factor kappa B
PC: protein carbonyl
PI: principal investigator
RAVLT: Rey Auditory Verbal Learning Test
RONS: reactive oxygen and nitrogen species
SPIRIT: Standard Protocol Items: Recommendations for Interventional Trials
STAT-3: signal transducer and activator of the transcription-3
TNF-α: tumor necrosis factor α
TRF: tocotrienol-rich fraction

## Data Availability

The data and materials are available upon request, subject to reasonable conditions.
